# Die nationale Begleitgesetzgebung zum Genehmigungsverfahren klinischer Prüfungen mit Humanarzneimitteln im Rahmen der Verordnung (EU) Nr. 536/2014

**DOI:** 10.1007/s00103-022-03629-5

**Published:** 2022-12-16

**Authors:** Marion Frech, Florian Dexel, Margareta Burgard, Yvonne Seibel

**Affiliations:** 1 Paul-Ehrlich-Institut, Referat S4 Rechtsangelegenheiten, Paul-Ehrlich-Str. 51–59, 63225 Langen, Deutschland; 2 Referat Z16 Justitiariat, Bundesinstitut für Arzneimittel und Medizinprodukte, Bonn, Deutschland; 3 Stabstelle P14 Fachrecht, Bundesinstitut für Arzneimittel und Medizinprodukte, Bonn, Deutschland

**Keywords:** Registrierung von Ethik-Kommissionen, Elektronisches Antragsverfahren, Zuständigkeiten, Freisetzungsgenehmigung, Nichteinwilligungsfähige Erwachsene, Registration of ethics committees, Electronic application procedure, Responsibilities, Deliberate release authorisation, Adults incapable of giving consent

## Abstract

Die Verordnung (EU) Nr. 536/2014 (Clinical Trials Regulation [CTR]) über klinische Prüfungen mit Humanarzneimitteln stellt nicht nur die Sponsoren, sondern auch die Behörden und Ethik-Kommissionen vor Herausforderungen. So waren grundlegende strukturelle Veränderungen für die Etablierung des neuartigen Genehmigungsverfahrens erforderlich. Die dazu notwendige Begleitgesetzgebung wurde 2016 mit dem Vierten Gesetz zur Änderung arzneimittelrechtlicher und anderer Vorschriften (4. AMGÄndG) sowie 2017 mit der Klinische Prüfung-Bewertungsverfahren-Verordnung (KPBV) geschaffen.

Das 4. AMGÄndG sah Änderungen des Arzneimittelgesetzes (AMG) vor, die bereits vor Geltungsbeginn der CTR in Kraft getreten sind. Ganz wesentlich betrifft das die Einführung einer Registrierungspflicht für Ethik-Kommissionen beim Bundesinstitut für Arzneimittel und Medizinprodukte (BfArM). Daneben enthält das 4. AMGÄndG Regelungen, die parallel zum Geltungsbeginn der CTR Anfang 2022 in Kraft getreten sind. Die Verordnung über die Anwendung der guten klinischen Praxis bei der Durchführung von klinischen Prüfungen mit Arzneimitteln zur Anwendung an Menschen (GCP-Verordnung) wurde aufgehoben. Der 6. Abschnitt des AMG über den Schutz des Menschen bei klinischen Prüfungen wurde neu gefasst, um die Vorgaben der CTR zu ergänzen und den Verfahrensablauf in das nationale Rechtssystem einzupassen. Insbesondere betrifft das Regelungen über die Zusammenarbeit zwischen Bundesoberbehörden und Ethik-Kommissionen, wie zu Zuständigkeiten, Fristen, Verfahrensabläufen und auch Gebühren. Auch wurden Regelungen für das nationale Verfahren der Freisetzungsgenehmigung bei klinischen Prüfungen mit Arzneimitteln, die gentechnisch veränderte Organismen (GVO) enthalten, getroffen sowie besondere Schutzvorschriften für spezielle Teilnehmergruppen geschaffen.

## Abstract (en)

The Regulation (EU) No 536/2014 (Clinical Trials Regulation [CTR]) on clinical trials on medicinal products for human use is a challenge not only for sponsors but also for national authorities and ethics committees. Thus, fundamental structural changes were necessary for the establishment of the novel authorisation procedure. The necessary accompanying law was created in 2016 with the Fourth Law on the Amendment of the Medicinal Products Act (4th AMGÄndG) and in 2017 with the Clinical Trial Assessment Procedure Ordinance (KPBV).

The 4th AMGÄndG provided for legal amendments to the Medicinal Products Act (AMG) that came into force in advance of the date of application of the CTR. Essentially, this concerns the introduction of a registration obligation for ethics committees at the Federal Institute for Drugs and Medical Devices (BfArM). In addition, the 4th AMGÄndG contains regulations that came into force at the beginning of 2022, in parallel with the date of application of the CTR. The Regulation on the application of Good Clinical Practice in the conduct of clinical trials of medicinal products for human use (GCP-Regulation, GCP-V) was repealed. The 6th section of the AMG on the protection of humans in clinical trials has been rewritten to supplement the requirements of the CTR and to fit the procedural flow into the national legal system. In particular, this concerns the regulations on the cooperation between higher federal authorities and ethics committees, such as responsibilities, deadlines, procedures and fees. Regulations were also made for the national procedure of release authorisation for clinical trials with medicinal products containing or consisting of genetically modified organisms (GMOs) as well as special protective regulations for special groups of participants.

## Einleitung

### Grundlagen

Die Verordnung (EU) Nr. 536/2014 ([1]; Clinical Trials Regulation, im Folgenden: CTR) über klinische Prüfungen mit Humanarzneimitteln und zur Aufhebung der Richtlinie 2001/20/EG [2] hat das seit 2004 etablierte System der Genehmigung von klinischen Prüfungen in Europa zumindest im Verfahrensablauf wesentlich verändert.

Ziel der CTR ist eine weitgehende Harmonisierung des Genehmigungsverfahrens von klinischen Prüfungen, die bei multinationalen klinischen Prüfungen durch ein gemeinsames europäisches Bewertungsverfahren erreicht wird. Gleichzeitig bleibt es bei der bisherigen Systematik, dass die rechtliche Entscheidung über den Genehmigungsantrag den nationalen Behörden obliegt. Weitere Kernpunkte der CTR sind die elektronische Kommunikation über das neu entwickelte EU-Portal und die komplexen Bearbeitungsprozesse und kürzeren Fristen, die alle Beteiligten vor eine große Herausforderung stellen [3].

### Bedarf an Begleitgesetzgebung

Die CTR ist unmittelbar geltendes Recht in der EU, so dass es keiner Umsetzung in das nationale Recht der Mitgliedstaaten (MS) bedarf. Ein Regelungsspielraum für die MS ist nur an wenigen Stellen vorhanden. Die detaillierten Regelungen des Sechsten Abschnitts des Arzneimittelgesetzes (AMG; §§ 40 bis 42b AMG) und die GCP-Verordnung (GCP‑V^1^; [4]) werden damit spätestens mit Ablauf der Übergangsfristen des Artikels 98 der CTR am 31.01.2025 ihren Anwendungsbereich fast vollständig verlieren. Das Vierte Gesetz zur Änderung arzneimittelrechtlicher und anderer Vorschriften (4. AMGÄndG) vom 20.12.2016 (Bundesgesetzblatt – BGBl. I S. 3048) verändert daher den Sechsten Abschnitt des AMG grundlegend und hebt die GCP‑V auf. Die Übergangsvorschrift des § 148 Absatz 1 und 2 AMG sichert die zeitweilige Fortgeltung des alten Rechts. Für den kleinen Bereich klinischer Prüfungen mit Arzneimitteln, die nicht in den Anwendungsbereich der CTR fallen, bestimmt § 148 Absatz 3 AMG die Fortgeltung des AMG und der GCP‑V in der bisherigen Fassung bis zum 23.12.2029.^2^

*Artikel 1 Nr. 8* des 4. AMGÄndG zur Einfügung der §§ 41a bis 41c AMG mit *sofortigem Inkrafttreten zum 24.12.2016*^3^ diente der frühzeitigen Etablierung eines Systems in Deutschland, welches den neuen Anforderungen der CTR an die Ethik-Kommissionen (EK) gerecht wird. *Artikel 2 *des 4. AMGÄndG, dessen *Inkrafttreten zum 27.01.2022*^4^ an den Geltungsbeginn der CTR angepasst war, enthält die CTR ergänzende Regelungen materiell-rechtlicher Art.

Im Folgenden werden 4 Bereiche, die in der Praxis der Bundesoberbehörden (BOB) von besonderer Relevanz sind, genauer vorgestellt:

1. das Registrierungsverfahren für EK beim Bundesinstitut für Arzneimittel und Medizinprodukte (BfArM) nach § 41a AMG;
2. die Regelungen zur Zusammenarbeit zwischen BOB und EK;
3. die Regelung zu klinischen Prüfungen mit einem Arzneimittel, das aus einem gentechnisch veränderten Organismus (GVO) oder einer Kombination von GVO besteht oder solche enthält (GVO-haltige Arzneimittel);
4. die nationalen Schutzvorschriften für besondere Teilnehmergruppen.

## 1. Registrierungsverfahren für Ethik-Kommissionen beim BfArM

An dem Verfahren zur Bewertung von klinischen Prüfungen nach der CTR dürfen gemäß § 41a AMG nur EK teilnehmen, die nach Landesrecht für die Prüfung und Bewertung klinischer Prüfungen zuständig sind und durch das BfArM im *Einvernehmen* mit dem Paul-Ehrlich-Institut (PEI) registriert worden sind. Ergänzend hierzu sieht § 41b Absatz 1 Satz 1 AMG die Ermächtigung für das Bundesministerium für Gesundheit (BMG) für den Erlass einer Rechtsverordnung vor, in der unter anderem die Einzelheiten zum Registrierungsverfahren der EK bestimmt werden sollen.

Sowohl § 41a als auch § 41b AMG sind schon am 24.12.2016 in Kraft getreten,^5^ um die Registrierung von EK vor dem Geltungsbeginn der CTR durchführen zu können. Auf der Grundlage des § 41b Absatz 1 AMG wurde am 12.07.2017 die Klinische Prüfung-Bewertungsverfahren-Verordnung (KPBV; [5]) erlassen, deren §§ 1–4 und 13 sowie Anlagen 1 und 2 am 15.07.2017 in Kraft getreten sind.

Die Einzelheiten des Registrierungsverfahrens sind in § 2 KPBV geregelt. Um die in § 2 Satz 1 KPBV vorgegebene elektronische Antragstellung zu ermöglichen, hat das BfArM 2017 ein voll elektronisches Verfahren über die Online-Umfrage-Applikation LimeSurvey (LimeSurvey GmbH, Hamburg, Deutschland) eingerichtet. Ziel war es, das Registrierungsverfahren von der Antragstellung bis zur Erstellung des Registrierungsbescheides angesichts der Digitalisierungsbestrebungen der CTR elektronisch zu gestalten (siehe Infobox 1).

### Infobox 1 Kommunikation betreffend das Registrierungsverfahren für Ethik-Kommissionen beim Bundesinstitut für Arzneimittel und Medizinprodukte (BfArM)

- Die Kommunikation erfolgt über die E‑Mail-Adresse: Registrierung-EK@bfarm.de
- Über diese E‑Mail-Adresse kann ein Zugangscode zur LimeSurvey-Anwendung beantragt und Änderungen können mitgeteilt werden
- Bekanntmachung des BfArM vom 20.07.2017 (Bundesanzeiger vom 04.08.2017) mit Verweisen zur Form und den Inhalt des Antrages auf seine Internetseite, auf der sich Erläuterungen zur Antragstellung sowie ein Test-Antragsformular befinden (https://www.bfarm.de/DE/Arzneimittel/Klinische-Pruefung/Ethik-Kommissionen/_node.html)

Die Registrierung soll nach der Gesetzesbegründung zu § 41a AMG bei einer von der für die Genehmigung klinischer Prüfungen unabhängigen Arbeitseinheit erfolgen.^6^ In Umsetzung dieser Vorgabe wird das Registrierungsverfahren im BfArM im Referat Justitiariat durchgeführt, das der zentralen Verwaltungsabteilung angehört und das ansonsten nicht mit fachrechtlichen Fragen zum Genehmigungsverfahren von klinischen Prüfungen befasst ist. Nach § 41a Absatz 3 AMG sind für eine Registrierung eine Reihe an Voraussetzungen durch geeignete Unterlagen nachzuweisen (siehe Infobox 2). Nähere Einzelheiten dazu enthält § 3 KPBV, dem als Anlagen Formulare für die Erklärungen zur antragsbezogenen und zur jährlichen Unabhängigkeitserklärung beigefügt sind.

### Infobox 2 Registrierungsvoraussetzungen für Ethik-Kommissionen nach § 41a Absatz 3 Arzneimittelgesetz (AMG)

1. Die erforderliche aktuelle wissenschaftliche Expertise der Mitglieder sowie der externen Sachverständigen
2. Die interdisziplinäre Zusammensetzung der Ethik-Kommission unter Beteiligung von je mindestens einem Juristen, einer Person mit wissenschaftlicher oder beruflicher Erfahrung auf dem Gebiet der Ethik in der Medizin, einer Person mit Erfahrung auf dem Gebiet der Versuchsplanung und Statistik, 3 Ärzten, die über Erfahrungen in der klinischen Medizin verfügen, davon ein Facharzt für klinische Pharmakologie und Toxikologie, sowie einem Laien
3. Der Ethik-Kommission gehören weibliche und männliche Mitglieder an und bei der Auswahl der Mitglieder und externen Sachverständigen werden Frauen und Männer mit dem Ziel der gleichberechtigten Teilhabe gleichermaßen berücksichtigt (dieser Punkt bereitet den Ethik-Kommissionen in der Praxisumsetzung Schwierigkeiten, ein starkes Bemühen, eine paritätische Besetzung zu erreichen, ist jedoch deutlich erkennbar)
4. Eine Geschäftsordnung, die insbesondere verpflichtende Regelungen zur Arbeitsweise der Ethik-Kommission trifft, dazu gehören insbesondere Regelungen zur Geschäftsführung, zum Vorsitz, zur Vorbereitung von Beschlüssen, zur Beschlussfassung sowie zur Ehrenamtlichkeit und Verschwiegenheitspflicht der Mitglieder und externen Sachverständigen
5. Eine Geschäftsstelle mit dem für die Organisation der Aufgaben der Ethik-Kommission erforderlichen qualifizierten Personal
6. Eine sachliche Ausstattung, die es ermöglicht, kurzfristig Abstimmungsverfahren durchzuführen und fristgerecht Stellungnahmen und Bewertungsberichte zu erstellen
7. Die Ethik-Kommission holt zu jedem Antrag Unabhängigkeitserklärungen der beteiligten Mitglieder und externen Sachverständigen ein, die beinhalten, dass diese keine finanziellen oder persönlichen Interessen, die Auswirkungen auf ihre Unparteilichkeit haben könnten, haben

Liegen die Voraussetzungen vor, genehmigt das BfArM im Einvernehmen mit dem PEI die Registrierung. Ein Ermessen ist insoweit nicht eingeräumt; es handelt sich vielmehr um eine gebundene Entscheidung.

Um den EK Zeit für eine ggf. erforderliche Anpassung ihrer Rechtsgrundlagen und Verfahrensabläufe an die detaillierten Vorgaben des AMG einzuräumen, hatte das BfArM die Registrierung zunächst auch unter Auflagen erteilt, die bis zum Geltungsbeginn der CTR erfüllt sein mussten. Je nach Organisation der Ethik-Kommission waren dazu Änderungen der Geschäftsordnung bzw. Satzung erforderlich, die zunächst durch andere Stellen genehmigt werden mussten. Zum Teil waren auch Änderungen der entsprechenden Landesgesetze erforderlich, was aufgrund der Legislativprozesse der Länder in Einzelfällen einige Zeit in Anspruch nahm.

Eine Liste der registrierten EK ist nach § 41a Absatz 6 AMG im Bundesanzeiger zu veröffentlichen. In der Bekanntmachung vom 16.12.2021 werden 37 EK aufgeführt.^7^ Diese Liste ist auch über die Internetseite des BfArM erreichbar^8^ und wird regelmäßig aktualisiert. Änderungen der Registrierungsvoraussetzungen sind gemäß § 41a Absatz 4 AMG unverzüglich dem BfArM mitzuteilen, das dann prüft, ob die Registrierungsvoraussetzungen weiterhin vorliegen.

Das BfArM kann im Einvernehmen mit dem PEI das Ruhen der Registrierung anordnen oder die Registrierung aufheben, wenn bekannt wird, dass die Voraussetzungen zur Registrierung nicht oder nicht mehr vorliegen, oder wenn ein Verstoß gegen die Verfahrensordnung (KPBV) vorliegt, § 41a Absatz 5 AMG. Die Anordnung des Ruhens der Registrierung kommt bei einem voraussichtlich nur vorübergehenden Wegfall der Voraussetzungen in Betracht. Die EK beantragen in der Regel selbst das Ruhen der Registrierung, wenn es z. B. Engpässe bei der personellen Besetzung gibt und deshalb ggf. Fristen nicht eingehalten werden könnten.

Für den Fall, dass nicht (mehr) ausreichend EK registriert sind, um die Bearbeitung der Verfahren nach der CTR sicherzustellen, wird das BMG in § 41c AMG ermächtigt, durch Rechtsverordnung eine „Bundes-Ethik-Kommission“ beim BfArM und beim PEI einzurichten. Bisher musste von dieser Ermächtigung kein Gebrauch gemacht werden.

## 2. Regelungen zur Zusammenarbeit zwischen Bundesoberbehörden und Ethik-Kommissionen

Ein wesentlicher Unterschied zum bisherigen Genehmigungsverfahren ist die Regelung in Artikel 8 Absatz 1 Unterabsatz 2 der CTR, die von *einer einzigen* nationalen Entscheidung jedes betroffenen MS über den Antrag auf Genehmigung einer klinischen Prüfung mit Arzneimitteln ausgeht. In den bisher im AMG etablierten Verfahren war einerseits eine Entscheidung der zuständigen BOB über den Genehmigungsantrag und andererseits separate Entscheidungen der oft mehreren zuständigen EK der Länder im Bewertungsverfahren der klinischen Prüfung vorgesehen. Es galt daher, diese bislang voneinander unabhängigen Verwaltungsverfahren in einem Verfahren zusammenzuführen.

Die Grundlage dafür hat das 4. AMGÄndG geschaffen. Darunter fallen insbesondere die Zuständigkeitsregelungen für die BOB und die registrierten EK, die wesentlichen Grundzüge der Zusammenarbeit von BOB und EK im Genehmigungsverfahren in § 40 AMG sowie die Rechtsgrundlage für eine diesbezügliche Verfahrensordnung in § 41b Absatz 1 AMG (siehe oben).

### Zuständigkeiten

Während die BOB nach § 40 Absatz 4 Satz 1 AMG für die Prüfung der in Teil I zu behandelnden Aspekte des Bewertungsberichts zuständig sind, prüfen die nach Landesrecht gebildeten und nach Bundesrecht registrierten EK nach § 40 Absatz 5 AMG die in Teil II des Bewertungsberichts zu behandelnden Aspekte. Soweit § 40 Absatz 4 Satz 2 AMG eine Stellungnahme der zuständigen EK zu einzelnen in Teil I des Bewertungsberichts zu behandelnden Aspekten vorsieht, hat die BOB diese Stellungnahme maßgeblich zu berücksichtigen, darf aber unter Beachtung der Vorgaben in § 40 Absatz 8 Satz 3 AMG davon abweichen. Diese Abgrenzung der Zuständigkeiten beachtet die getrennten Verwaltungsräume von Bund und Ländern und genügt dem sog. Verbot der Mischverwaltung.

### Ablauf des Genehmigungsverfahrens nach der Klinische Prüfung-Bewertungsverfahren-Verordnung (KPBV)

Wie bereits angesprochen, enthält § 41b Absatz 1 Satz 1 AMG die Ermächtigung für das BMG, mit Zustimmung des Bundesrates eine Rechtsverordnung zu erlassen, in der die Zusammenarbeit zwischen den BOB und den registrierten EK bei der Bearbeitung von Genehmigungsanträgen für klinische Prüfungen, die sich nach der CTR richten, geregelt wird (siehe zu den Inhalten Infobox 3).

#### Infobox 3 Wesentliche Inhalte der Klinische Prüfung-Bewertungsverfahren-Verordnung (KPBV) des Bundesministeriums für Gesundheit (BMG) zur Regelung der Zusammenarbeit zwischen den Bundesoberbehörden und den registrierten Ethik-Kommissionen gemäß § 41b Absatz 1 Satz 2 Arzneimittelgesetz (AMG)

- Einzelheiten zum Registrierungsverfahren der Ethik-Kommissionen
- Gebühren- und Rahmensätze der registrierten Ethik-Kommissionen für ihre Mitwirkung bei den Genehmigungsverfahren
- Kriterien für den Geschäftsverteilungsplan der Ethik-Kommissionen, einschließlich der Faktoren für die Verteilung der Anträge, sowie
- Zuständigkeitsregeln für das Ersuchen um zusätzliche Informationen beim Sponsor nach der Verordnung (EU) Nr. 536/2014

Umgesetzt wurde dieser gesetzliche Auftrag mit der *KPBV*. So wie die Regelungen des 4. AMGÄndG teilweise erst mit Geltungsbeginn der CTR in Kraft traten, sind auch die Regelungen des Vierten Abschnitts (§§ 5–11) KPBV zum Genehmigungsverfahren erst am 31.01.2022 in Kraft getreten, § 13 Absatz 2 KPBV. Die bereits im AMG grundlegend vorgegebenen Abläufe des neuen Verwaltungsverfahrens werden darin hinsichtlich der Zusammenarbeit von BOB und registrierten EK konkretisiert. Im Wesentlichen geht es dabei um Fristen und Zuständigkeitszuweisungen für einzelne Verfahrenshandlungen. § 5 regelt die Validierung des Antrags, § 6 betrifft die Prüfung der nach Artikel 6 der CTR in Teil I des Bewertungsberichts und § 7 die nach Artikel 7 der CTR in Teil II des Bewertungsberichts zu behandelnden Aspekte. In § 8 finden sich die Regelungen zur Zusammenarbeit von BOB und EK bei Hinzufügung eines weiteren MS der Europäischen Union. § 9 KPBV regelt die Einzelheiten der Zusammenarbeit zum Verfahren bei wesentlichen Änderungen einer klinischen Prüfung, § 11 das Verfahren bei notwendigen Korrekturmaßnahmen gemäß § 42 Absatz 2–4 AMG.

### Bewertung von Sicherheitsaspekten klinischer Prüfungen

§ 10 KPBV betrifft das Bewertungsverfahren nach Artikel 44 der CTR. Artikel 44 Absatz 2 Satz 1 der CTR legt lediglich fest, dass die Mitgliedstaaten bei der Bewertung von Sicherheitsaspekten nach den Artikeln 42 und 43 der CTR zusammenarbeiten. Artikel 44 Absatz 2 Satz 2 und 3 der CTR sehen für die Regelung der Einzelheiten dieser Zusammenarbeit den Erlass von Durchführungsrechtsakten vor. Zum Zeitpunkt des Erlasses des 4. AMGÄndG sowie der KPBV existierte noch kein solcher Durchführungsrechtsakt. § 40c Absatz 2 AMG, der die Einbindung der EK in dieses Bewertungsverfahren festlegt, sowie § 10 KPBV, der die Übermittlung der Bewertung gemäß § 40c Absatz 2 AMG von der EK an die BOB vorsieht, konnten daher die Einzelheiten der Zusammenarbeit noch nicht detailliert regeln.

Der entsprechende Durchführungsrechtsakt, die Durchführungsverordnung (EU) 2022/20 [6], ist rechtzeitig am 30.01.2022 zum Geltungsbeginn der CTR in Kraft getreten und gilt seit dem 31.01.2022.^9^ Soweit erforderlich, z. B. im Hinblick auf bestimmte Bearbeitungsfristen, könnten daher nun Konkretisierungen zur Zusammenarbeit zwischen BOB und EK zur Sicherheitsbeurteilung in § 10 KPBV ergänzt werden.

### Fristen für die Ethik-Kommissionen

Ein wesentlicher Diskussionspunkt im Verordnungsgebungsverfahren zur KPBV waren die Fristen, die den EK für ihre Prüfung eingeräumt wurden. Die einzelnen Bearbeitungsfristen in der KPBV mussten sich an den kurzen Fristen der europäischen CTR orientieren und sich in das zeitlich vorgegebene Verfahren einfügen. Insofern ergab sich die Schwierigkeit, die Bedürfnisse sowohl der EK als auch der BOB angemessen zu berücksichtigen. Einerseits sollte den EK im nationalen Austausch mit den BOB für ihre Bewertung ausreichend Zeit eingeräumt werden. Andererseits sollte aber der jeweils zuständigen BOB genügend Zeit gelassen werden, um sich je nach Rolle im europäischen Verfahren mit anderen MS oder dem Sponsor auszutauschen, die Bewertungen der zuständigen EK in das Verfahren einzubeziehen und die nach der europäischen CTR festgelegten Verfahrenshandlungen rechtzeitig vornehmen zu können. Der Bundesrat hat in seiner Sitzung vom 07.07.2017 die Entschließung gefasst, die Bundesregierung um einen Erfahrungsbericht 2 Jahre nach Inkrafttreten der §§ 5–12 KPBV zu bitten.^10^ Darin soll insbesondere auch zur Zusammenarbeit zwischen den BOB und den registrierten EK nach der KPBV sowie zur Angemessenheit der Fristen aus der CTR im Hinblick auf eine für den Probandenschutz ausreichende Prüftiefe des Antrags berichtet werden.

### Entscheidung über die klinische Prüfung

Nach § 40 Absatz 1 AMG darf mit einer klinischen Prüfung nur begonnen werden, wenn die zuständige BOB sie nach Artikel 8 der CTR genehmigt hat. Gemäß § 40 Absatz 8 Satz 1 AMG übermittelt die zuständige BOB die Entscheidung über das EU-Portal an den Sponsor. Bei der Entscheidung der zuständigen BOB nach Artikel 8 Absatz 1 der CTR kann es sich um eine Genehmigung, eine Genehmigung unter Auflagen oder eine Versagung der Genehmigung handeln. Der Tenor der Entscheidung folgt dabei aus den Ergebnissen der Bewertungsberichte. Die Entscheidung nach Artikel 8 der CTR bleibt verfahrensrechtlich, anders als beispielsweise die Entscheidung über eine zentrale Arzneimittelzulassung nach der Verordnung (EG) Nr. 726/2004 [7], eine nationale Entscheidung. Soweit die CTR keine Regelungen trifft, gelten die nationalen Regelungen des Verwaltungsverfahrens- und -prozessrechts, z. B. im Hinblick auf die Rechtsbehelfsbelehrung, einzelne Formvorschriften, Rechtsbehelfsfristen oder zum Rechtsweg.

### Gebühren

Ebenfalls neu zu regeln war das Verfahren der Gebührenerhebung, da nach Artikel 87 der CTR ein MS für die Bewertung eines Antrags nie mehrere Zahlungen an unterschiedliche an der Bewertung des Antrags beteiligte Stellen verlangen darf. Zuvor hatten die BOB und die EK für ihre Bewertungen in den getrennten Verfahren jeweils gesondert und unabhängig voneinander Gebühren erhoben.

Die Vorgabe der CTR wurde in § 40 Absatz 6 AMG umgesetzt. Danach erhebt die zuständige BOB eine Gesamtgebühr im Sinne der Artikel 86 und 87 der CTR. Die Gesamtgebühr beinhaltet also die Gebühr der EK und die Gebühr der BOB; der Gebührenschuldner erhält nur einen Gebührenbescheid.

Die Gebühren, die den EK für ihre Leistungen zustehen, sind in der Anlage 3 (zu § 12) KPBV aufgelistet. Die zuständige EK erhebt gemäß § 40 Absatz 6 AMG eine Gebühr für die Bearbeitung eines Antrags nach Maßgabe der KPBV und teilt diese der zuständigen BOB mit. Die Erhebung der Gebühr durch die EK erfolgt nicht gegenüber dem Gebührenschuldner [8], sondern sie ist eher im Sinne einer Berechnung der Gebühr zu verstehen.^11^

In Bezug auf diese Gebühr gelten für die zuständigen EK im Übrigen die gebührenrechtlichen Regelungen der Länder, z. B. für Ermäßigungen. Dies kann zur Folge haben, dass Ermäßigungen der Gebühr der EK und der Gebühr der BOB in unterschiedlichem Ausmaß gewährt werden.

Die für die Leistungen der BOB nach der CTR vorgesehenen Gebühren finden sich in Abschnitt 5 der Besonderen Gebührenverordnung des BMG (BMGBGebV; [9]), die am 01.10.2021 in Kraft getreten ist. Die Gebühren der EK sind als Auslagen gemäß Nr. 12.1 der Tabelle 1 bzw. Nr. 4.3 der Tabelle 2 des Abschnittes 5 BMGBGebV durch die BOB mit zu erheben.

Die BOB überweisen gemäß § 12 Absatz 3 KPBV den vereinnahmten Betrag innerhalb von 21 Tagen nach seiner Vereinnahmung und nach Eintritt der Bestandskraft des Gebührenbescheides über die Gesamtgebühr an den Träger der zuständigen EK.

Aufgrund des Systems der nachgelagerten Gebührenerhebung liegen noch keine Erfahrungen diesbezüglich vor.

## 3. Arzneimittel mit gentechnisch veränderten Organismen (GVO)

Bei der Durchführung klinischer Prüfung am Menschen mit GVO-haltigen Arzneimitteln sind nicht nur die Vorschriften des Arzneimittelrechts zu beachten. Da die Verwendung von solchen Arzneimitteln in einer klinischen Prüfung eine absichtliche Freisetzung von GVO in die Umwelt darstellt, unterliegt sie auch der europäischen Richtlinie 2001/18/EG [10], der sog. Freisetzungsrichtlinie. Diese Freisetzungsrichtlinie ist in Deutschland im Wesentlichen in das Gentechnikrecht umgesetzt worden – allerdings mit Ausnahme des Bereichs der Anwendung von GVO am Menschen (vgl. § 2 Absatz 3 Gentechnikgesetz (GenTG; [11]).

Für den Bereich der Verwendung von GVO-haltigen Arzneimitteln im Rahmen einer klinischen Prüfung wurden stattdessen Regelungen im Arzneimittelrecht geschaffen, um die Freisetzungsrichtlinie in nationales Recht umzusetzen. Genauer gesagt sind bislang die Vorgaben des Teils B der Freisetzungsrichtlinie über die „absichtliche Freisetzung von GVO zu anderen Zwecken als dem Inverkehrbringen“ in das Regelungswerk über die Genehmigung klinischer Prüfungen eingegliedert gewesen. So enthielten die §§ 40 ff. AMG a. F.^12^ und vor allem die GCP‑V spezielle Vorschriften für GVO-haltige Prüfpräparate im regulatorischen Umfeld der klinischen Prüfung.

### Integrierte Freisetzungsgenehmigung

Wesentliches Merkmal des Genehmigungsverfahrens von klinischen Prüfungen mit GVO-haltigen Arzneimitteln war bisher die sog. integrierte Freisetzungsgenehmigung, d. h., die Genehmigung der klinischen Prüfung umfasste die Genehmigung der Freisetzung des GVO im Rahmen der klinischen Prüfung (§ 9 Absatz 4 Satz 3 GCP‑V a. F.). Dazu sah das AMG a. F. bzw. die GCP‑V eine spezielle Umweltverträglichkeitsprüfung (Environmental Risk Assessment – ERA) durch die BOB in Zusammenarbeit mit dem Bundesamt für Verbraucherschutz und Lebensmittelsicherheit (BVL) vor, um zu überprüfen, ob von einem GVO-haltigen Prüfpräparat unvertretbare schädliche Auswirkungen auf die Gesundheit Dritter (z. B. das Studienpersonal oder Angehörige) und die Umwelt (z. B. durch Abfälle des Prüfpräparats, Ausscheidungen der teilnehmenden Personen) zu erwarten sind. Die Entscheidung über die Freisetzung traf die nach § 77 AMG zuständige BOB im *Benehmen* mit dem BVL in einem einheitlichen Bescheid mit der Entscheidung über den Antrag auf Genehmigung der klinischen Prüfung. § 42 Absatz 3 Satz 2 Nr. 3 i. V. m. § 40 Absatz 1 Satz 3 Nr. 2a AMG a. F. enthielt dazu einen eigenen Versagungsgrund.

Diese integrierte Freisetzungsgenehmigung bot den Sponsoren klinischer Prüfungen mit GVO-haltigen Arzneimitteln den Vorteil, dass sie sich nicht bei einer weiteren Behörde separat um eine Freisetzungsgenehmigung auf Basis der gentechnikrechtlichen Vorschriften bemühen mussten.

### Kein Bestandteil der Verordnung (EU) Nr. 536/2014

Die CTR beinhaltet – wie auch bisher die Richtlinie 2001/20/EG – selbst keine Regelungen zu GVO-haltigen Prüfpräparaten. Artikel 91 der CTR stellt vielmehr klar, dass die Richtlinie 2001/18/EG durch diese Verordnung vollumfänglich ihre Geltung behält.

Die nationale Begleitgesetzgebung zur CTR musste daher so gestaltet werden, dass die nach der Richtlinie 2001/18/EG erforderlichen regulatorischen Vorgaben für die Freisetzung von GVO im Rahmen einer klinischen Prüfung auch bei klinischen Prüfungen nach der CTR eingehalten werden. Materiell-rechtlich wurden dafür mit Artikel 2 des 4. AMGÄndG die bewährten Regelungen des AMG a. F. und der GCP‑V für GVO-haltige Prüfpräparate soweit erforderlich in den neuen Sechsten Abschnitt des AMG eingepflegt. So führt z. B. § 40 Absatz 7 AMG^13^ die zusätzlichen Antragsunterlagen auf, die neben dem Antragsdossier gemäß dem Anhang I der CTR vorzulegen sind; § 40d AMG fasst die besonderen Mitteilungs‑/Informationspflichten des Prüfers, des Sponsors und der Bundesoberbehörde zusammen. Der spezielle Versagungsgrund im Falle von unvertretbaren schädlichen Auswirkungen der klinischen Prüfung mit einem GVO-haltigen Arzneimittel für die Gesundheit Dritter oder die Umwelt hat seinen Platz in § 40a Satz 1 Nr. 4 AMG gefunden (Tab. 1).

**Table 1:** 

Altes Recht	Neues Recht	Inhalt
*AMG alte Fassung*	*AMG neue Fassung*	–
§ 40 Absatz 1 Satz 3 Nr. 2a	§ 40a Satz 1 Nr. 4	Versagungsgrund
*GCP‑V*		
§ 7 Absatz 4 Nr. 3	§ 40 Absatz 7 Satz 1, 2	Antragsunterlagen
§ 9 Absatz 4 Satz 3	§ 40 Absatz 7 Satz 3, 4	Zusammenarbeit mit dem Bundesamt für Verbraucherschutz und Lebensmittelsicherheit (BVL) und integrierte Freisetzungsgenehmigung
§ 10 Absatz 1 Satz 1 Nr. 5	§ 40c Absatz 3	Zustimmungspflichtige Änderungen
§ 11 Absatz 2	§ 40d Nr. 1	Maßnahmen zum Schutz vor unmittelbarer Gefahr
§ 12 Absatz 7	§ 40d Nr. 2	Unterrichtungspflicht des Prüfers
§ 13 Absatz 7 und 9a	§ 40d Nr. 3 und 4	Mitteilungspflichten des Sponsors
§ 14 Absatz 6	§ 40d Nr. 5	Unterrichtungspflichten der Bundesoberbehörden

*GCP‑V* Verordnung über die Anwendung der guten klinischen Praxis bei der Durchführung von klinischen Prüfungen mit Arzneimitteln zur Anwendung an Menschen

Auch wurden aus der GCP‑V die bewährte Benehmensregelung mit dem BVL und das Konzept der integrierten Freisetzungsgenehmigung übernommen. Der Bescheid der BOB mit der Entscheidung über die klinische Prüfung nach Artikel 8 Absatz 1 der CTR i. V. m. § 40 Absatz 8 AMG enthält hierzu bei GVO-haltigen Prüfpräparaten sowohl die positive oder negative Entscheidung über die klinische Prüfung gemäß den Bewertungsverfahren der CTR (siehe oben) als auch die positive oder negative Entscheidung über die Freisetzung des GVO im Rahmen dieser klinischen Prüfung nach den nationalen Kriterien.

### Herausforderungen in der praktischen Anwendung

Eine ganz wesentliche Neuerung für die Beantragung und Bearbeitung von Anträgen im Zusammenhang mit klinischen Prüfungen nach der CTR ist das EU-Portal. Erst Jahre nach der Verkündung des 4. AMGÄndG 2016 wurde es fertig gestellt und der Gesamtumfang der Funktionen von der Europäischen Arzneimittel-Agentur (EMA) festgelegt. Im Ergebnis hat sich herausgestellt, dass die Inhalte des EU-Portals streng an den Geltungsbereich der CTR gekoppelt sind. Daher können die nach der Freisetzungsrichtlinie gesetzlich notwendigen ERA-Unterlagen dort nicht als Teil der Antragsunterlagen zu der dazugehörigen klinischen Prüfung hochgeladen werden, sondern sind separat national einzureichen. Diese Trennung setzt sich auch im Verfahrensablauf fort: Die Verfahrensregelungen der CTR sehen nicht vor, dass formelle und inhaltliche Mängelfragen zu den ERA-Unterlagen im Ablauf des europäischen Bewertungsverfahrens gestellt und berücksichtigt werden. Die BOB werden daher die ERA-Bewertung, einschließlich der Benehmensherstellung mit dem BVL und der Zusammenarbeit mit der EK, außerhalb des EU-Portals auf nationaler Ebene organisieren.

Hieraus können sich Konstellationen im Verwaltungsverfahren entwickeln, in denen sich die beiden Verfahrensteile zeitlich auseinanderentwickeln. Denn mitunter wird der Antrag auf Freisetzungsgenehmigung nach dem AMG zum Zeitpunkt der nach Artikel 8 Absatz 1 der CTR erforderlichen Mitteilung der Entscheidung über die klinische Prüfung gemäß den Vorgaben der CTR noch nicht entscheidungsreif sein. Sofern nicht über Nebenbestimmungen die erforderliche Entscheidungsreife hergestellt werden kann, wird die zuständige BOB die Entscheidung über die Freisetzung in einem gesonderten Bescheid treffen. Es bleibt aber wie bisher dabei, dass die klinische Prüfung trotz einer Genehmigung der klinischen Prüfung nach der CTR nicht begonnen werden darf, solange keine Genehmigung der Freisetzung des GVO durch die BOB vorhanden ist.

## 4. Schutzvorschriften für besondere Teilnehmergruppen

Nach § 40a Satz 1 Nr. 2 AMG ist auch weiterhin die Teilnahme von Personen an einer klinischen Prüfung ausgeschlossen, die auf gerichtliche oder behördliche Anordnung in einer Anstalt untergebracht sind. Als zusätzliche nationale Maßnahme lässt Artikel 34 der CTR dies zu.

Auch die Voraussetzungen für die Teilnahme Minderjähriger an klinischen Prüfungen haben sich durch die Regelungen in den Artikeln 28, 29 und 32 der CTR nicht wesentlich geändert. Bereits mit dem Zwölften Gesetz zur Änderung des Arzneimittelgesetzes vom 30.07.2004 (BGBl. I S. 2031) ist die Möglichkeit der gruppennützigen^14^ klinischen Prüfung von Arzneimitteln bei Minderjährigen in das AMG aufgenommen worden. Um unter Geltung der CTR das bisherige Schutzniveau für Minderjährige beizubehalten, hat der deutsche Gesetzgeber von dem Regelungsspielraum des Artikels 29 Absatz 8 der CTR Gebrauch gemacht. So sieht § 40b Absatz 3 Satz 1 AMG als weitere Voraussetzung die auch bisher in § 40 Absatz 4 Nr. 3 Satz 4 AMG a. F. vorgesehene zusätzliche eigene Einwilligung des Minderjährigen vor, soweit er in der Lage ist, das Wesen, die Bedeutung und die Tragweite der klinischen Prüfung zu erkennen und seinen Willen hiernach auszurichten. Insgesamt wird somit unter Geltung der CTR durch die Ergänzungen im AMG ein identisches Schutzniveau bei klinischen Prüfungen in der vulnerablen Personengruppe der Minderjährigen erreicht.

### Teilnahme von nichteinwilligungsfähigen Erwachsenen

Umstritten und intensiv diskutiert war hingegen auf politischer, gesellschaftlicher und fachlicher Ebene die Frage der Teilnahme nichteinwilligungsfähiger Erwachsener an gruppennützigen klinischen Prüfungen. Die in Deutschland auch historisch begründete zurückhaltende bis ablehnende Haltung in dieser äußerst sensiblen Frage stand der Forderung und dem Bedarf nach weiterer Forschung für neue Arzneimittel, z. B. für dementielle Erkrankungen, gegenüber. Nach den bisherigen Regelungen des AMG war eine solche Konstellation anders als bei Minderjährigen ausgeschlossen.

Artikel 31 Absatz 1 Buchstabe g) ii) der CTR lässt die Teilnahme nichteinwilligungsfähiger Erwachsener an gruppennützigen klinischen Prüfungen zwar zunächst unter engen Voraussetzungen zu. Artikel 31 Absatz 2 der CTR lässt aber wiederum Raum für den Erlass strengerer nationaler Regelungen, die die Durchführung solcher klinischen Prüfungen verbieten.

Der am 27.11.2015 veröffentlichte Referentenentwurf^15^ zum 4. AMGÄndG sah in § 40b Absatz 4 AMG-Entwurfsfassung noch ein solches Verbot vor. Danach war die Teilnahme nichteinwilligungsfähiger Erwachsener ausschließlich an klinischen Prüfungen mit Arzneimitteln erlaubt, die einen direkten Nutzen für die betroffene Person erwarten lassen, der die Risiken und Belastungen der Teilnahme überwiegt. Dies entsprach nach der Begründung des Referentenentwurfs unter anderem dem Beschluss des Bundestages vom 31.01.2013^16^, der die Beibehaltung des bisherigen Schutzniveaus verlangte.

Der Gesetzentwurf der Bundesregierung vom 11.03.2016 des 4. AMGÄndG rückte von diesem Verbot ab und ließ die Durchführung solcher klinischen Prüfungen in § 40b Absatz 4 Satz 2 AMG-Entwurfsfassung erstmals unter der Voraussetzung zu, dass eine entsprechende Patientenverfügung gemäß § 1901a Absatz 1 Satz 1 Bürgerliches Gesetzbuch dies gestattet.^17^

Nach dem letztlich verabschiedeten 4. AMGÄndG ist gemäß § 40b Absatz 4 Satz 2 ff. AMG die Durchführung gruppennütziger klinischer Prüfungen mit nichteinwilligungsfähigen Erwachsenen nunmehr möglich. Die Voraussetzungen sind im Vergleich zum Gesetzesentwurf noch verschärft worden. So ist im weiteren Verlauf des Gesetzgebungsverfahrens insbesondere das Erfordernis einer ärztlichen Aufklärung auch für eine durch eine Festlegung vorweggenommene Einwilligung in eine spätere gruppennützige klinische Prüfung in die Regelung aufgenommen worden.^18^ Nach Satz 3 muss der Betreuer zusätzlich prüfen, ob die Festlegungen, die der seinerzeitigen Einwilligung der nicht mehr einwilligungsfähigen Person zugrunde lagen, auf die aktuelle Situation noch zutreffen.

## Fazit

Mit dem 4. AMGÄndG hat der Gesetzgeber es geschafft, den nationalen Rechtsrahmen in umfassender Weise frühzeitig an die neuen Anforderungen aus der Verordnung (EU) Nr. 536/2014 (Clinical Trials Regulation [CTR]) anzupassen. Bis zum Geltungsbeginn der CTR wurden 37 Ethik-Kommissionen registriert, so dass von der Ermächtigung zur Einrichtung einer Bundes-Ethik-Kommission kein Gebrauch gemacht werden musste. Ob sich die in § 40 AMG und den §§ 5–12 KPBV festgelegten Regeln für die Zusammenarbeit der BOB mit den EK in der Praxis bewähren werden, wird sich erst zukünftig zeigen.

Für klinische Prüfungen mit GVO-haltigen Arzneimitteln bleiben die bewährten materiellen Regelungen zur Umsetzung der Freisetzungsrichtlinie erhalten. Der Verfahrensablauf erhält jedoch wegen des von der CTR ausgegrenzten Rechtsbereichs der Freisetzungsrichtlinie eine separate nationale Komponente, ohne das Konzept der integrierten Freisetzungsgenehmigung aufzugeben.

Hinsichtlich der inhaltlichen Anforderungen an klinische Prüfungen mit besonders schutzbedürftigen Personengruppen ist das bisherige Schutzniveau weitestgehend erhalten geblieben. Eine Ausnahme stellen die gruppennützigen klinischen Prüfungen mit nichteinwilligungsfähigen Erwachsenen dar, die nun erstmalig unter sehr engen Voraussetzungen erlaubt werden.

## Footnotes

1 GCP-V: Verordnung über die Anwendung der guten klinischen Praxis bei der Durchführung von klinischen Prüfungen mit Arzneimitteln zur Anwendung an Menschen.

2 Dies betrifft „nicht industrielle“ Gewebezubereitungen und hämatopoetische Stammzellzubereitungen i. S. d. Richtlinie 2004/23/EG des Europäischen Parlaments und des Rates vom 31.03.2004 zur Festlegung von Qualitäts- und Sicherheitsstandards für die Spende, Beschaffung, Testung, Verarbeitung, Konservierung, Lagerung und Verteilung von menschlichen Geweben und Zellen (Amtsblatt d. Europäischen Union [ABl.] L 102 vom 07.04.2004, S. 48) sowie Blutzubereitungen i. S. d. Artikels 3 Nummer 6 der Richtlinie 2001/83/EG des Europäischen Parlaments und des Rates vom 06.11.2001 zur Schaffung eines Gemeinschaftskodexes für Humanarzneimittel (ABl. L 311 vom 28.11.2001, S. 67) im Zuständigkeitsbereich des Paul-Ehrlich-Instituts.

3 Artikel 1 Nr. 8 i. V. m. Artikel 13 Absatz 1 des 4. AMGÄndG.

4 Artikel 2 i. V. m. Artikel 13 Absatz 2 des 4. AMGÄndG i. d. F. des Artikels 4 Nr. 3 des Gesetzes zum Erlass eines Tierarzneimittelgesetzes und zur Anpassung arzneimittelrechtlicher und anderer Vorschriften vom 27.09.2021 (BGBl. I S. 4530).

5 Siehe Fußnote 3.

6 Bundestagsdrucksache (BT-Drs.) 18/8034, S. 37.

7 Bundesanzeiger amtlicher Teil (BAnz AT) 19.01.2022 B7.

8 https://www.bfarm.de/DE/Arzneimittel/Klinische-Pruefung/Ethik-Kommissionen/_node.html, abgerufen am 14.06.2022.

9 Vgl. Artikel 15 der Durchführungsverordnung (EU) 2022/20.

10 BR-Drs. 405/17 (Beschluss vom 07.07.2017).

11 Vgl. BT-Drs. 18/8034, S. 44.

12 a. F. = in der Fassung vom 26.01.2022.

13 Sofern nicht gesondert mit a. F. gekennzeichnet, ist im Folgenden das AMG i. d. F. ab dem 27.01.2022 gemeint.

14 Eine gruppennützige klinische Prüfung liegt vor, wenn die Teilnahme an der klinischen Prüfung nur einen erwarteten Nutzen für die repräsentierte Bevölkerungsgruppe hat, zu der der Teilnehmer gehört, nicht jedoch einen direkten Nutzen für ihn selbst, vgl. Artikel 31 Absatz 1 Buchstabe g) Ziffer ii) der CTR.

15 https://www.bundesgesundheitsministerium.de/fileadmin/Dateien/3_Downloads/Gesetze_und_Verordnungen/GuV/A/151125_RefE_4.AMG_Novelle.pdf.

16 BT-Drs. 17/12183.

17 BR-Drs. 120/16.

18 BT-Drs. 18/10235.
